# The Radial Bulging and Axial Strains of Intervertebral Discs during Creep Obtained with the 3D-DIC System

**DOI:** 10.3390/biom12081097

**Published:** 2022-08-10

**Authors:** Mengying Yang, Dingding Xiang, Song Wang, Weiqiang Liu

**Affiliations:** 1 Department of Mechanical Engineering, Tsinghua University, Beijing 100084, China; 2 Tsinghua Shenzhen International Graduate School, Tsinghua University, Shenzhen 518055, China; 3 Biomechanics and Biotechnology Lab, Research Institute of Tsinghua University in Shenzhen, Shenzhen 518057, China; 4 State Key Laboratory of Tribology, Tsinghua University, Beijing 100084, China; 5 School of Mechanical Engineering and Automation, Northeastern University, Shenyang 110819, China

**Keywords:** creep, intervertebral disc, bulge, strain, 3D-DIC

## Abstract

Creep-associated changes in disc bulging and axial strains are essential for the research and development of mechano-bionic biomaterials and have been assessed in various ways in ex vivo creep studies. Nonetheless, the reported methods for measurement were limited by location inaccuracy, a lack of synchronousness, and destructiveness. To this end, this study focuses on the accurate, synchronous, and noninvasive assessment of bugling and strains using the 3D digital image correlation (3D-DIC) system and the impact of creep on them. After a preload of 30 min, the porcine cervical discs were loaded with different loads for 4 h of creep. Axial strains and lateral bulging of three locations on the discs were synchronously measured. The three-parameter solid model and the newly proposed horizontal asymptote model were used to fit the acquired data. The results showed that the load application reduced disc strains by 6.39% under 300 N, 11.28% under 400 N, and 12.59% under 500 N. Meanwhile, the largest protrusion occurred in the middle of discs with a bugling of 1.50 mm, 1.67 mm, and 1.87 mm. Comparison of the peer results showed that the 3D-DIC system could be used in ex vivo biomechanical studies with reliability and had potential in the assessment of the mechanical behavior of novel biomaterials. The phenomenon of the largest middle protrusion enlightened further the strength of spinal implants in this area. The mathematical characterizations of bulging and strains under different loads yielded various model parameters, which are prerequisites for developing implanted biomaterials.

## 1. Introduction

Creep is the progressive deformation of the disc that occurs under constant load and stress below the fracture threshold [1]. In the human body, the creep of intervertebral discs (IVDs) causes 1 to 2 cm [2] or a 1.1% difference [3] in normal heights, which is mainly attributed to the loss and gain of the disc height [4,5]. The disc height loss is often accompanied by lateral bulging, which is the major cause of disc protrusion and herniation [6]. It is also of importance as providing valuable data for further preclinical evaluations of spinal implants and biomechanical experiments of disc injuries.

Thus far, several studies have investigated disc bulging in in vitro creep tests in various ways. Heuer et al. [7,8,9] developed a laser-based three-dimensional contour scanner that was mounted in a spine tester to measure disc bulging during creep, providing a high-precision disc bulging assessment with complicated and customized mechanical structures. Another similar study reported by Fewster et al. [10] included applying the 3D laser scanner with several steel pins inserted into the disc to align the 3D surface profiles from each functional spinal unit and estimate the curvature of the endplate for acquiring bulges. Their previous results [11] showed that the proposed system achieved errors of less than 0.2 mm. As this approach was destroyed, they may result in inaccurate data. Dupré et al. [12,13] adopted an optoelectrical tracking system to quantify a disc bulge after nucleus fortification for treating a degenerated nucleus. The results showed that a significant reduction in posterior, lateral, and posterolateral disc bulges was observed during lateral bending for the fortified treatment compared with disc-only. Pei et al. [14] applied five red oil points on the surface of specimens to mark the outer annular surface and two orthogonally placed cameras to capture images. Subsequently, photographic data were extracted using video editing software, and the markers were digitized using image processing software with a seven-pixel brush tool. The horizontal and vertical differential pixel numbers were computed as the radial and axial deformations. Though easily to be conducted, their method was limited in less automated processes.

As common imaging methods in clinics, computerized tomography (CT) and magnetic resonance imaging (MRI) can also be adopted in in vitro measurements. Mengoni et al. [15] used CT images to measure the disc bulge successfully, though with radiation and high costs. During specimen preparation, CT-compatible markers were embedded into the surfaces of the endcaps. Additionally, forty glass fiducial markers were attached to the surface of each disc using petroleum jelly. Coordinates for all glass markers on the CT images were automatically calculated to obtain the bulge data using image processing and recognition. MRI images were mainly used to acquire shape and texture features in in vivo measurements. Mayerhoefer et al. [16], Hung et al. [17], and Sundarsingh et al. [18] made efforts in the analysis of the parameter sensitivity. Their studies confirmed that texture features and geometric parameters were sensitive to posterior disc bulging and, thus, can be used as quantitative biomarkers that predict disease. To improve the accuracy, Beulah et al. [19] further proposed a computer-aided diagnostic system, which was based on a new segmentation method, to identify the disc bulge in axial lumbar spine T2-weighted MR images. On applying the proposed system, an accuracy of 92.78% was obtained. Lao et al. [20] also compared whether adding flexion and extension to the traditional neutral views would be beneficial in the diagnosis of cervical disc bulges in an MRI study. The results showed that a significant increase in the degree of cervical disc bulge was found by examining the extension views, thus confirming that the kinematic MRI views provided valuable added information, especially in situations where symptomatic radiculopathy was present without any abnormalities demonstrated on traditional neutral MRI.

To sum up, those methods mentioned above did have limitations in acquiring disc bulge data in in vitro practice. Fortunately, the digital image correlation (DIC) provided a new method to measure deformations with outcomes of high accuracy and noncontact, through which images of an object can be obtained in digital forms for further analysis [21]. In the early 1990s, the DIC technique was extended to stereovision systems, designating them 3D-DIC, which employed two or more cameras to record digital images of a tested object from multiple viewpoints. By using DIC to perform a cross-camera subset matching, a calibrated stereovision system obtains the true and 3D positions of each point on a nonplanar object. Over recent years, the 3D-DIC has seen remarkable growth, with applications in aerospace [22,23], micro-scale measurements [24], biomaterials [25], and fracture mechanics [26,27,28]. In this study, a commercial 3D-DIC system has been adopted to measure the axial strains and disc bulging of compressed discs under physiological creep successfully.

The purpose of this study was to investigate time-dependent disc bulging and axial strains as a cause of physiological creep using the 3D-DIC system. The relations between lateral bulging and axial strains were also revealed. Additionally, a new mathematical model for fitting disc bulging was also proposed. The results showed that the new model had high quality, and the relevant model parameters under different magnitudes of creep loads were calculated. The numerical model provided approximations of the physiological bulging of discs, thus facilitating further comparisons of mechanical properties, especially lateral deformation, between biomaterials and natural discs.

## 2. Methods

### 2.1. System Setup and Calibration

**Figure 1:**
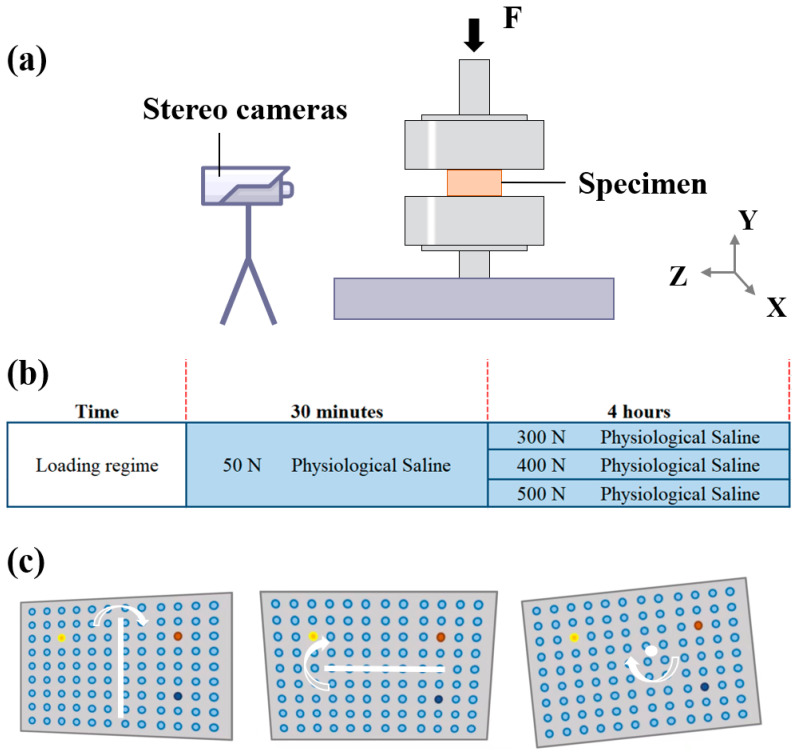
(**a**) Physical setup for stereovision measurements using stereovision cameras. The specimen was tested under prolonged axial compression, and the synchronized images were acquired by all cameras. (**b**) Loading regime. (**c**) Acquisition of calibration images with grid rotation about three axes.

The physiological loads were axially applied with a universal testing machine (MTS-CMT 6104, MTS System (China) Co., Ltd., Shenzhen, China) and was registered at 1 Hz. The custom 3D-DIC system (VIC-3D LS-2M, Correlated Solutions Inc., Columbia, SC, USA) was adopted for measuring bulging and strains at 0.5 Hz, which included stereovision cameras (with a resolution of 1920 × 1200 for each pixel size of 5.86 μm) and postprocessing software (Vic3D). The camera system was set in front of the testing machine (Figure 1a). The specimens were firstly exposed to 50 N axial compression for 30 min for reducing water. Subsequently, the formal creep tests were carried out under three different magnitudes of loads (300 N, 400 N, and 500 N) with a loading rate of 5 N/s for 4 h (Figure 1b), which was reported to be sufficient by Paul et al. [29]. The loading regime in this study was according to the earlier ex vivo study, conducted by O’Connell et al. [30] and Yang et al. [31,32], who adopted 4 h as the loading duration to quantify the time–response during creep. Five specimens were tested under the same conditions at room temperature; after which, the results were averaged.

Before the tests, the calibration process was needed, taking images of the calibration grid in a range of orientations that included tilting the grid forward, backward, and rotating (Figure 1c). Since the calibration grid was rigid, these images could be used to perform a shape measurement of the grid, thus determining the camera model and system parameters. The whole system should not be moved during or after calibration. The error of the 30 calibration image pairs should be less than 0.100, indicating that the cameras were successfully calibrated. Through the comparison algorithm based on the calibration images and the random speckle patterns during the tests, subregions from the “deformed” and “undeformed” images were compared, thus generating a full field of sensor plane measurements.

### 2.2. Specimen Preparation

Ten porcine cervical spinal segments (C2–7) were used in this experiment. Soft tissue and facet capsules of the specimens were removed, and adjacent vertebral bodies were removed ~3.5 mm from the disc with a hand saw. All specimens were stored at −20 °C in the refrigerator and used within a week. Before testing, the discs were thawed overnight in saline solution to promote rehydration.

**Figure 2:**
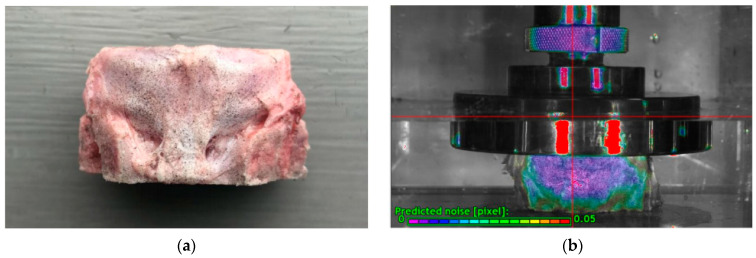
(**a**) Example of the speckle pattern created by matte black ink with high contrast and random speckles. (**b**) The speckle pattern was recognized by the software with a low level of noise.

The surfaces of the discs were prepared with matte speckles before being tested (Figure 2a). In order to provide good tracking information, the speckles should ideally be 3–5 pixels (about 0.6 mm) in size to optimize the resolution. The speckles were recognized in the following data processing algorithms to compute the disc bulging and strains (Figure 2b).

### 2.3. Disc Bulging and Strains Computation

During the creep tests, the speckles on the specimen surfaces were captured by the cameras and could be evaluated by means of image processing methods. Since the system parameters were determined during the calibration, it was possible to determine X, Y, and Z displacements and the corresponding strains by measuring the speckles distance and displacement. In principle, these deformations were determined by correlating surface image data from the calibration images and the deformed specimen images.

Disc strains were defined as the strain expressed along the virtual extensometer from the top of the disc to the bottom, which was an average value and the green-Lagrange strain. Therefore, the extensometer should be located within the disc region and could be evaluated in both X and Y directions, named exx and eyy. Only eyy values were considered in this study. Disc bulging was computed by stereovision algorithms, and three points were equidistantly assigned from the upper part of the disc to the lower.

Since the specimens needed to be submerged in liquid during the tests, the refraction that occurred at the interfaces was inevitable, producing significant errors in the measurements. To avoid the refractive error, the integrated algorithm of the Variable Ray Origin (VRO) camera model was used. This enabled the system to eliminate measurement bias when imaging through optical interfaces.

### 2.4. Fitting Models of Axial Strains and Lateral Bulging

During the creep period, the strain–time data were curve-fitted using the Kelvin function (Equation (1)). The model was typically used to characterize the creep behavior of natural IVDs [33,34,35].

(1) $$\varepsilon \left(t\right)=\frac{\delta_{0}}{E_{1}}+\frac{\delta_{0}}{E_{2}}\left(1-e^{-\frac{t}{\tau }}\right)$$

**Figure 3:**
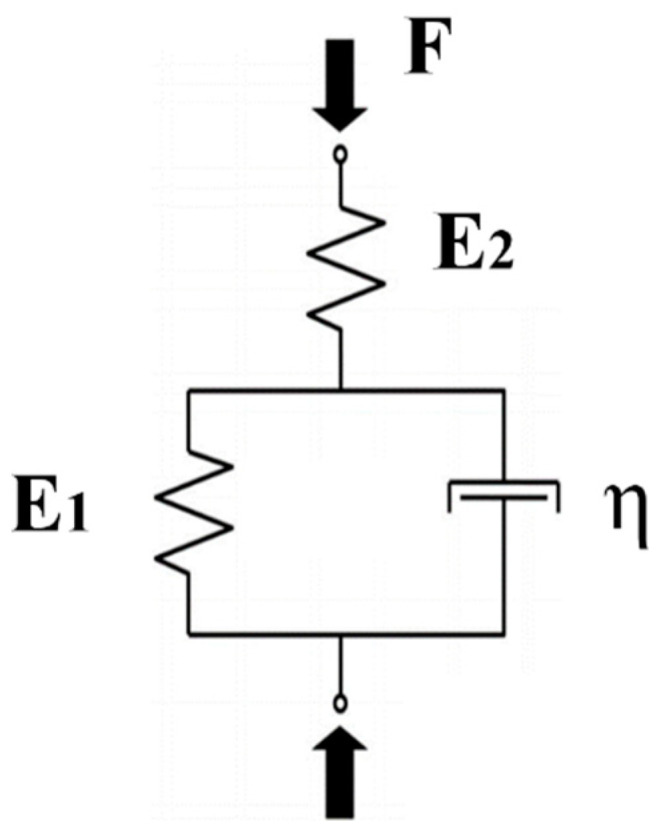
The Kelvin model used for simulating compressive creep behavior of natural discs.

The Kelvin model describes a linear elastic body connected with an ideal viscous body in parallel and then connected with a linear elastic body in a series (Figure 3). ε is the strain of the disc, $E_{1}$ is the viscous (equilibrium) modulus, $E_{2}$ is the elastic (instantaneous) modulus, $\delta_{0}$ is the stress applied on the disc, t is the elapsed time, and *τ* is the time constant. η is the viscosity and can be derived from $\eta =E_{2}\times \tau$. The curve-fitting procedure used the nonlinear least-squares fitting method to minimize the summed squares of residuals in MATLAB (MathWorks, Natick, MA, USA, version 19.2).

There was no suitable mathematical model used for describing disc bulging during the creep. The protrusion of the disc increases with prolonged time but is limited by structure, making bulging constant under the same load. Therefore, we proposed the parametric horizontal asymptote equation (Equations (2)) for fitting bulging. Their fitting precision R^2^ were computed. The bulging data of three different points (upper, middle, and lower) were computed and fitted using the nonlinear least-squares method in MATLAB (MathWorks, Natick, MA, USA, version 19.2).


(2) $$x\left(t\right)=\frac{a*t^{2}}{b+c*t^{2}}$$

a, b, and c are the model parameters, and x is the bulging value at the time, t.

### 2.5. Statistical Analysis

Differences between the groups (loads) were determined using a one-way analysis of variance. The level of significance was set at *p* = 0.05. Both variance and correlation analyses were performed in SPSS v26.0 (SPSS, Chicago, IL, USA).

## 3. Results

### 3.1. Effects of Loads on Disc Strains

**Figure 4:**
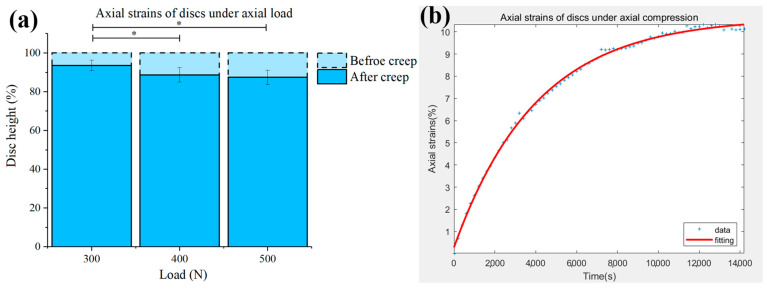
(**a**) Axial strains of discs under compression of 300 N, 400 N, and 500 N. The error bar reflected the standard deviation. * Denotes significance between groups (*p* < 0.05). (**b**) Typical creep results accompanied by its fitted curve from Equation (1).

An axial compression of 300 N resulted in a disc strain of 6.39% (±2.61) and increased with the applied load from 11.28% (±3.56) to 12.59% (±3.68) under 400 N and 500 N (Figure 4a). The typical strain results were fitted by Equation (1) with good quality (Figure 4b).

The associated parameters of Equation (1) are shown in Table 1. $\sigma_{0}$ was derived from $\sigma_{0}=\frac{F}{A}$, where F was the applied load (300 N, 400 N, and 500 N), and A was the cross-sectional area of the porcine disc obtained from the literature (~800 mm^2^) [36]. 

### 3.2. Effects of Loads on Disc Bulging

**Figure 5:**
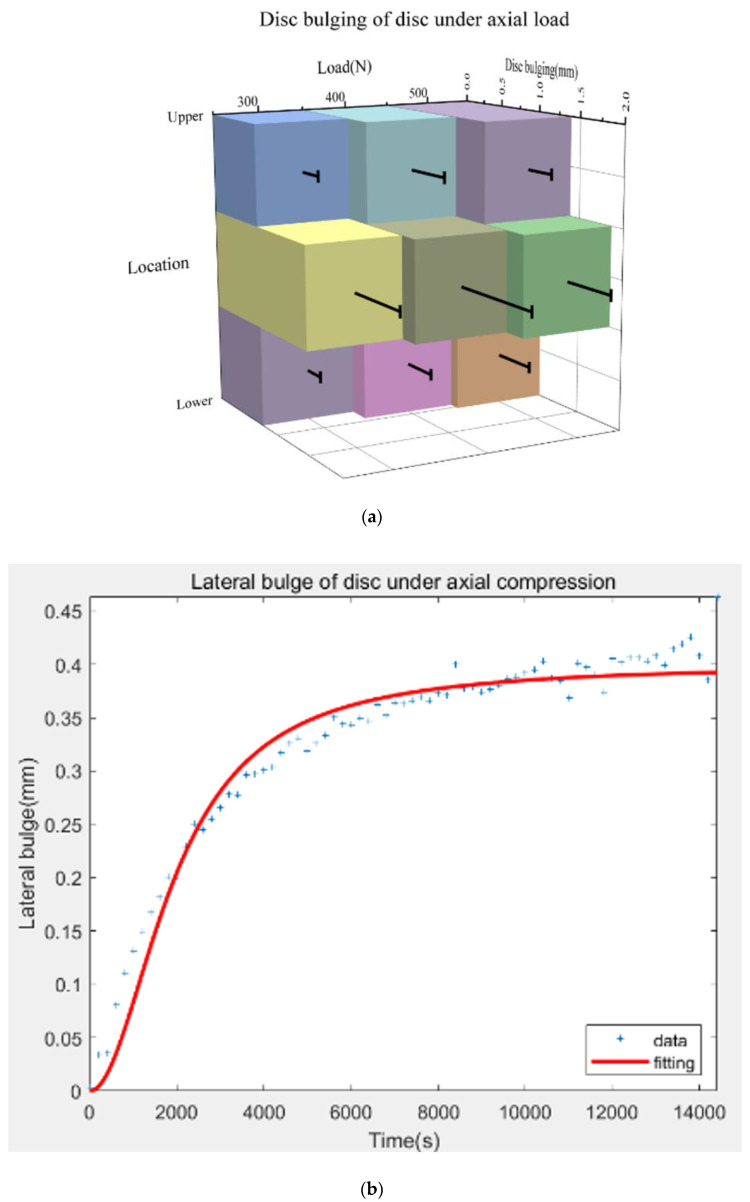
(**a**) Disc bulging of three different locations under compressions of 300 N, 400 N, and 500 N. The error bar reflects the standard deviation. Data were presented as the means ± standard deviations. (**b**) Typical disc bulging results accompanied by its fitted curve from Equation (2).

An axial compression of 300 N induced a bulging of 0.74 (±0.20) mm, 1.50 (±0.61) mm, and 0.76 (±0.24) mm in the upper, middle, and lower part of the discs. The maximum outward disc bulging occurred in the middle part under different magnitudes of loads and increased from 1.67 (±0.83) mm to 1.87 (±0.48) mm under 400 N and 500 N (Figure 5a). The typical bulging results were fitted by Equation (2) with good quality (Figure 5b).

The fitting precision (R^2^) was determined by both the equation itself and the data quality. Based on the results, the new model had high fitting precision (Table 2). Additionally, the bulging of the discs in the present study was comparable to previous studies (Table 3).

### 3.3. Relations between Disc Strains and Bulging

**Figure 6:**
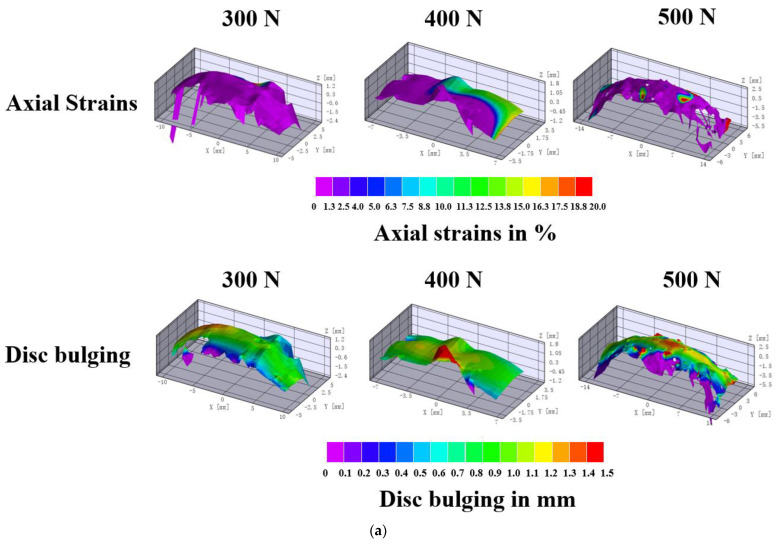

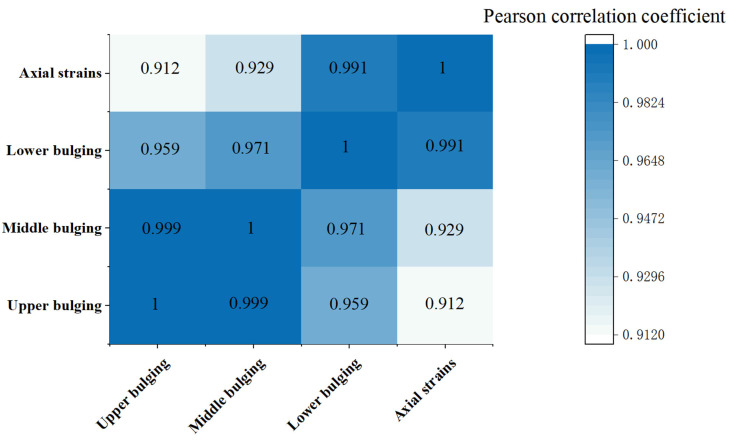
(**a**) Disc strains and bulging maps of the frontal surfaces. The surface maps summarized the single-axial loading under compressions of 300 N, 400 N, and 500 N. The data could be extracted from every part or point of these maps. The blank areas were due to the poor imaging quality at this location and were not recorded. (**b**) The relation between disc strains and bulging (upper, middle, and lower parts of the disc). It was considered that two factors have a strong correlation when R (the absolute value of the correlation) > 0.8; R 0.3–0.8 was considered a weak relation; R < 0.3 was considered no correlation.

The strains and bulging maps (Figure 6a) showed the distribution of deformation on the disc surface. With increasing the load, the disc strains and bulging grew larger. An axial load of 500 N produced the largest disc strains and outward bulging, while the maximum protrusion occurred in the middle of the disc under every load. 

Although water outflowed during the creep, the value of the strain and bulging seemed to be positively correlated. Herein, we used the Pearson correlation coefficient to analyze the relations of the upper, middle, and lower bulging with the disc strains and the relations between every bulging. The results revealed a strong relationship between the bulging and strain and also within the bulging of each part (Figure 6b).

## 4. Discussion

Creep under axial compression is the main loading status of discs during physiological activities. The creep-associated axial strains and lateral bulging are affected by both the duration and magnitudes of the load. In a previous study, O’Connell et al. [40,41] reported that the measured disc height reduced 30% of the equilibrium displacement after creep for 4 h, thus suggesting that, by reducing the creep duration to 4 h in vitro, it would be enough to simulate the creep responses, as well as avoiding the decay of specimens. Thus, in this study, 4 h was adopted as our test duration and expected for assessing long-time physiological responses. According to the previous in vivo literature [42], 0.375–0.625 MPa, which was the range of compressive stress conducted by this study, was within the daily activity level in porcine discs. Axial deformation during the whole creep period was contributed by both the vertebral bodies and the cartilage endplates when using bone–disc–bone specimens; thus, they should be excluded to obtain an accurate assessment [7,33,43]. Accordingly, in this study, the 3D-DIC system was adopted to accurately measure the axial strains and lateral bulging of only the disc area for discarding interferences that soft tissues and bony structures brought. This method was proved to be effective in in vitro biomechanical tests by O’Connell et al. [40], who adopted Vic2D (Vic2D, Correlated Solutions Inc.) to evaluate the two-dimensional internal displacements and average strains of discs after 20 min under 1000 N compression. Gullbrand et al. [44] used a high-resolution digital camera (A3800; Basler, Exton, PA, USA) and a custom MATLAB program to track the axial displacements of two ink marks placed on each vertebral body adjacent to the disc in their study. Except for the failure in radial data acquisition and invasive preconditioning, the axial data from their study could be merely extracted from the two preprinted marks, and other areas of the disc could not be selected. The 3D-DIC system adopted in this study was superior in data acquisition from three dimensions, including the radial (X and Z axes) and axial deformation (Y axis), and from every point in the area of interest.

In Figure 4a, the disc height reduced 6.39% (±2.61) under 300 N, 11.28% (±3.56) under 400 N, and 12.59% (±3.68) under 500 N. It seemed that the reduction of the disc height was not proportional to the applied load, and this can be attributed to the fluid transfer during creep. From the perspective of the viscoelasticity of IVDs, the loss of disc height under prolonged creep was mainly governed by the process of fluid outflow, while the water content in natural IVDs was limited, within the range of 70–80%. Combined with the osmotic pressure in the body fluid environment, the disc height will ultimately reach an equilibrium, during which the reduction of the disc height may not be constant [45,46].

It can be seen from Figure 5a that the largest bulging occurred in the middle part of discs during a prolonged period. This can be attributed to the constant volume compression of the nucleus pulposus (NP) in a semi-confined configuration. During the axial compression, the gelatinous and spherical NP was compressed by the loaded upper vertebral body and, thus, was extruded along the transverse plane, forcing the outer annulus fibers (AF) outward, similar to the shape of a drum. Moreover, this phenomenon provided the biomechanical basis for the clinical observation that the middle part of the IVD was the place where disc herniation and subsequent nerve compression mostly occurred. Future implanted biomaterials of the total disc replacement (TDR) could enhance the strength of the middle region of the prosthesis so as to eliminate the secondary protrusion. For instance, in the recent design of the polyvinyl alcohol-bacterial cellulose (PVA-BC) hydrogel-based biomaterials [47], a higher amount of BC can be added at the middle part of the composite to enhance the toughness.

Bovine discs [48] and porcine discs [49,50] were common big animal models used in in vitro studies due to the similarities to human discs and the lack of facet joints and, thus, can be compared directly to human discs. These tissues were also usually used in the preclinical assessment of IVD repair [51,52,53]. It can be seen from Table 3 that the bulging data in this study were comparable to other published literature. Compressions of 300 N, 400 N, and 500 N were selected by this study and by Reuber et al. [37], Heuer et al. [7,8,9], and Fewster et al. [10]. Longer in the loading durations, the bulging data in this study were relatively higher than those previously reported in the literature while on the same order of magnitude, indicating the feasibility of this 3D-DIC system in in vitro biomechanics. It should be noted that different loading protocols were used in above aggregated studies, resulting in a wide range of bulging data in Table 3.

For mathematical characterization, the Kelvin model was adopted for fitting axial strains data. As studies on bulging under creep are lacking, there was no suitable function for fitting bulging data. The empirical equation of horizontal asymptotes was therefore proposed in this study to facilitate mathematical fitting. All R^2^ of the model were higher than 0.9, indicating good fitting qualities of the equation. The proposed model could be used for further attempts of bulging fitting of biomaterials in mechanical tests. While it should be admitted that Equation (2) is purely empirical, further progress could be made towards developing functions with more physical significance. In the last few years, many researchers have studied the mechanism of disc responses after prolonged loading. The compression-induced herniation and the relation with the microstructure were studied by Sapiee et al. [54]. The results showed that shear differentially loaded the oblique counter-fibers in lateral annulus, thus increasing its vulnerability to disruption and herniation. From the perspective of chemo-mechanical coupling effects, both the osmotic pressure of the surrounding liquid [55], magnitudes of preloads [56], and loading rates [57] have coupling effects on the mechanical responses of IVDs. Feki et al. [55] also revealed that 18 h of unloading was still not enough to regain the original height of discs, either in hypo-osmotic, iso-osmotic, or in hyper-osmotic conditions. Recently, through scanning electron microscopy (SEM), Tavakoli et al. [58] firstly revealed the elastic fibers network that the straight and thick parallel fibers formed across the NP. Based on this, a novel structural model for the elastic fibers in the NP were proposed. With the rapid development of computational simulations, finite element modeling (FEM) with rational simplifications [59] can provide information on the underlying tissue that cannot be assessed from in vitro experiments [15,60] or original images [61]. Komeili et al. [62] and Castro et al. [63] systemically developed spinal models with linear elastic, hyper-elastic, and biphasic material constitutive models and applied various physiological conditions. Their studies confirmed the central role of the fluid pressure in spinal load sharing and highlighted the differences in energy distribution predicted by various models. As a major failure mode, the damage model in AF has been established to evaluate the fatigue responses [64] and interactions between the lamellar, interlamellar, and NP [65]. The outcome overturned the existing assumption of a uniform stress distribution in the AF and defined that the critical interlamellar spots had the highest delamination potential. Combined with the findings in this study, the area of spinal biomechanics will benefit from a more accurate and multiscale model, and the bio-inspired tissue-engineered scaffolds used for artificial IVDs could thus be developed.

## 5. Conclusions

In summary, the results from this study demonstrated that the 3D-DIC system could be used for measuring micro-deformations noninvasively and accurately. This provides a new method, combining stereo vision and image processing, in the area of biomechanical assessments. Additionally, this study helped us with understanding the disc responses under physiological loads, which have important clinical relevance. As the largest protrusion was observed at the middle part of the discs, it is necessary to reinforce IVD protheses at the middle part to avoid secondary herniation after implantation. Furthermore, the newly proposed model for lateral bulging fitting can facilitate future biomechanical studies and are prerequisites for developing implanted biomaterials and implants. Future studies of the mathematical characterization of bulging with more physical significance are warranted. The research and development of artificial disc prostheses with mechanic and structural mimicry are also urgently needed.

## Figures and Tables

**Table 1:** Associated parameters of the strains fitting model (Kelvin Model; Equation (1)).

Load	$\sigma_{0}\;(\mathrm{MPa})$	$E_{1}\;(\mathrm{MPa})$	$E_{2}\;(\mathrm{MPa})$	τ	η (MPa·s)	R^2^
300 N	0.375	8.361	0.196	7008.480	1373.662	0.9993
400 N	0.500	2.171	0.060	4056.267	243.376	0.9979
500 N	0.625	0.706	0.049	3379.265	165.584	0.9979

**Table 2:** Associated parameters and R^2^ of the bulging fitting model (Equation (2)).

Equation (2)	Location	a	b	c	R^2^
300 N	Upper	3.891	3.891 × 10^7^	16.62	0.9194
	Middle	7.859	9.956 × 10^7^	36.689	0.9056
	Lower	4.186	1.559 × 10^7^	24.033	0.9321
400 N	Upper	2.457	1.648 × 10^7^	5.823	0.9687
	Middle	2.897	4.757 × 10^7^	5.438	0.9220
	Lower	1.147	1.889 × 10^7^	2.624	0.9109
500 N	Upper	3.545	7.101 × 10^8^	12.905	0.9122
	Middle	8.968	2.897 × 10^7^	15.507	0.9543
	Lower	1.288	1.504 × 10^6^	5.138	0.9238

**Table 3:** Results of disc bulging from the literature compared with this study. In cases where numerical values were not available, estimates were obtained from the figures. In cases where healthy, degenerate, and treated IVDs were tested, the data from the healthy IVDs were recorded. In cases where different areas of IVDs were tested, the data from the largest part were recorded. It should be noted that the loading protocols of the literature were different from each other.

Reference	Specimen	Number	Load (N)	Time (min)	Bulging (mm)
Reuber et al., 1982 [37]	human lumbar	14	400	-	0.55
Wenger et al., 1997 [38]	human lumbar	16	2500	2.5	0.65 ± 0.42
Meakin et al., 2000 [39]	sheep lumbar	18	1000	-	0.277 ± 0.218
Heuer et al., 2007 [7]	human lumbar	7	500	15	1.1
Heuer et al., 2008 [8]	human lumbar	6	500	15	0.86
Heuer et al., 2012 [9]	human lumbar	6	500	15	0.8
Pei et al., 2013 [14]	ovine lumbar	15	1000	5	0.343 ± 0.141
Lao et al., 2014 [20]	human cervical	3000	in vivo	in vivo	C23-0.6
					C34-1.5
					C45-1.7
					C56-1.8
					C67-1.4
					C7T1-0.3
Dupré et al., 2016 [13]	human lumbar	25	250	-	0.4
Fewster et al., 2020 [10]	porcine cervical	12	10	15	1.27
			300		1.46
			600		1.43
			1200		1.45
Mengoni et al., 2021 [15]	bovine caudal	6	110	90	1.2
Our results	porcine cervical	10	300	240	1.50 ± 0.61
			400	240	1.67 ± 0.83
			500	240	1.87 ± 0.83

## References

[1] Twomey L., Taylor J. (1982). Flexion creep deformation and hysteresis in the lumbar vertebral column. Spine.

[2] Leatt P., Reilly T., Troup J.G. (1986). Spinal loading during circuit weight-training and running. Br. J. Sports Med..

[3] Tyrrell A.R., Reilly T., Troup J.D.G. (1985). Circadian variation in stature and the effects of spinal loading. Spine.

[4] McMillan D.W., Garbutt G., Adams M.A. (1996). Effect of sustained loading on the water content of intervertebral discs: Implications for disc metabolism. Ann. Rheum. Dis..

[5] Reilly T., Tyrrell A., Troup J.D.G. (1984). Circadian variation in human stature. Chronobiol. Int..

[6] Adams M.A., Hutton W.C. (1985). Gradual disc prolapse. Spine.

[7] Heuer F., Schmidt H., Schmidt H., Claes L., Wilke H.J. (2007). Creep associated changes in intervertebral disc bulging obtained with a laser scanning device. Clin. Biomech..

[8] Heuer F., Schmidt H. (2008). The relation between intervertebral disc bulging and annular fiber associated strains for simple and complex loading. J. Biomech..

[9] Heuer F., Schmidt H., Kafer W., Graf N., Wilke H.J. (2012). Posterior motion preserving implants evaluated by means of intervertebral disc bulging and annular fiber strains. Clin. Biomech..

[10] Fewster K.M., Noguchi M., Gooyers C.E., Wong A., Callaghan J.P. (2020). Exploring the regional disc bulge response of the cervical porcine intervertebral disc under varying loads and posture. J. Biomech..

[11] Fewster K.M., Haider S., Gooyers C.E., Callaghan J., Wong A. (2020). A computerised system for measurement of the radial displacement of the intervertebral disc using a laser scanning device. Comput. Methods Biomech. Biomed. Eng. Imaging Vis..

[12] Cuchanski M., Cook D., Whiting D.M., Cheng B.C. (2011). Measurement of occlusion of the spinal canal and intervertebral foramen by intervertebral disc bulge. SAS J..

[13] Dupré D.A., Cook D.J., Bellotte J.B., Oh M.Y., Whiting D., Cheng B.C. (2016). Disc nucleus fortification for lumbar degenerative disc disease: A biomechanical study. J. Neurosurg. Spine.

[14] Pei B.Q., Hui L., Li D.Y., Fan Y.B., Wang C., Wu S.Q. (2013). Creep bulging deformation of intervertebral disc under axial compression. Bio-Med. Mater. Eng..

[15] Mengoni M., Zapata-Cornelio F.Y., Wijayathunga V.N., Wilcox R.K. (2021). Experimental and computational comparison of intervertebral disc bulge for specimen-specific model evaluation based on imaging. Front. Bioeng. Biotechnol..

[16] Mayerhoefer M.E., Stelzeneder D., Bachbauer W., Welsch G.H., Mamisch T.C., Szczypinski P., Weber M., Peters N.H.G.M., Fruehwald-Pallamar J., Puchner S. (2012). Quantitative analysis of lumbar intervertebral disc abnormalities at 3.0 Tesla: Value of T2 texture features and geometric parameters. NMR Biomed..

[17] Hung I.Y.-J., Shih T.T.F., Chen B.-B., Guo Y.L. (2021). Prediction of Lumbar Disc Bulging and Protrusion by Anthropometric Factors and Disc Morphology. Int. J. Environ. Res. Public Health.

[18] Sundarsingh S., Kesavan R. (2020). Diagnosis of disc bulge and disc desiccation in lumbar MRI using concatenated shape and texture features with random forest classifier. Int. J. Imaging Syst. Technol..

[19] Beulah A., Sharmila T.S., Pramod V.K. (2018). Disc bulge diagnostic model in axial lumbar MR images using Intervertebral disc Descriptor (IdD). Multimed. Tools Appl..

[20] Lao L., Daubs M.D., Scott T.P., Phan K.H., Wang J.C. (2014). Missed cervical disc bulges diagnosed with kinematic magnetic resonance imaging. Eur. Spine J..

[21] Sutton M.A., Yan J.H., Tiwari V., Schreier H.W., Orteu J.J. (2008). The effect of out-of-plane motion on 2D and 3D digital image correlation measurements. Opt. Lasers Eng..

[22] Helm J.D., Sutton M.A., McNeill S.R. (2003). Deformations in wide, center-notched, thin panels: Part I: Three dimensional shape and deformation measurements by computer vision. Opt. Eng..

[23] Helm J.D., Sutton M.A., McNeill S.R. (2003). Deformations in wide, center-notched, thin panels: Part II: Finite element analysis and comparison to experimental measurements. Opt. Eng..

[24] Schreier H.W., Garcia D., Sutton M.A. (2004). Advances in light microscope stereo vision. Exp. Mech..

[25] Sutton M.A., Ke X., Lessner S.M., Goldbach M., Yost M., Zhao F., Schreier H.W. (2008). Strain field measurements on mouse carotid arteries using microscopic three-dimensional digital image correlation. J. Biomed. Mater. Res. A.

[26] Sutton M.A., Helm J.D., Boone M.L. (2001). Experimental study of crack growth in thin sheet 2024-T3 aluminum under tension–torsion loading. Int. J. Fract..

[27] Yan J.H., Sutton M.A., Deng X., Cheng C.S. (2007). Mixed mode fracture of ductile thin sheet materials under combined in-plane and out-of-plane loading. Int. J. Fract..

[28] Sutton M.A., Yan J.H., Deng X.M., Cheng C.S., Zavattieri P. (2007). 3D digital image correlation to quantify deformation and COD in ductile aluminum under mixed-mode I/III loading. Opt. Eng..

[29] Paul C.P.L., Schoorl T., Zuiderbaan H.A., van der Veen A.J., ban de Ven P.M., Mullender M.G. (2013). Dynamic and Static Overloading Induce Early Degenerative Processes in Caprine Lumbar Intervertebral Discs. PLoS ONE.

[30] O’Connell G.D., Jacobs N.T., Sen S., Vresilovic E.J., Elliott D.M. (2011). Axial Creep Loading and Unloaded Recovery of the Human Intervertebral Disc and the Effect of Degeneration. J. Mech. Behav. Biomed. Mater..

[31] Yang M.Y., Cui Y.Y., Zhang Y., Wu H.K., Hu B.B., Wang S., Liu W.Q. (2022). Quantitative Characterization of the Elasticity, Net Creep, and Swelling of the Intervertebral Disc: An In Vitro Experiment. J. Bionic Eng..

[32] Yang M.Y., Xiang D.D., Wang S., Liu W.Q. (2022). In Vitro Studies for Investigating Creep of Intervertebral Discs under Axial Compression: A Review of Testing Environment and Results. Materials.

[33] Keller T.S., Spengler D.M., Hansson T.H. (1987). Mechanical behavior of the human lumbar spine. I. Creep analysis during static compressive loading. J. Orthop. Res..

[34] Burns M.L., Kaleps I., Kazarian L.E. (1984). Analysis of compressive creep behavior of the vertebral unit subjected to a uniform axial loading using exact parametric solution equations of Kelvin-solid models–Part, I. Human intervertebral joints. J. Biomech..

[35] Kaleps I., Kazarian L.E., Burns M.L. (1984). Analysis of compressive creep behavior of the vertebral unit subjected to a uniform axial loading using exact parametric solution equations of Kelvin-solid models–Part II. Rhesus monkey intervertebral joints. J. Biomech..

[36] Showalter B.L., Beckstein J.C., Martin J.T., Beattie E.E., Elliott D.M. (2012). Comparison of Animal Discs Used in Disc Research to Human Lumbar Disc. Spine.

[37] Reuber M., Schultz A., Denis F., Spencer D. (1982). Bulging of lumbar intervertebral disks. J. Biomech. Eng..

[38] Wenger K.H., Schlegel J.D. (1997). Annular bulge contours from an axial photogrammetric method. Clin. Biomech..

[39] Meakin J.R., Hukins D.W.L. (2000). Effect of removing the nucleus pulposus on the deformation of the annulus fibrosus during compression of the intervertebral disc. J. Biomech..

[40] O’Connell G.D., Malhotra N.R., Vresilovic E.J., Elliott D.M. (2011). The Effect of Nucleotomy and the Dependence of Degeneration of Human Intervertebral Disc Strain in Axial Compression. Spine.

[41] O’Connell G.D., Johannessen W. (2007). Human internal disc strains in axial compression measured noninvasively using magnetic resonance imaging. Spine.

[42] Ekstrm L., Holm S., Holm A.K., Hansson T. (2004). In Vivo Porcine Intradiscal Pressure as a Function of External Loading. Clin. Spine Surg..

[43] Pollintine P., Tunen M.V., Luo J., Brown M.D., Dolan P., Adams M.A. (2010). Time-dependent Compressive Deformation of the Ageing Spine: Relevance to Spinal Stenosis. Spine.

[44] Gullbrand S.E., Ashinsky B.G., Martin J.T., Pickup S., Smith L.J., Mauck R.L., Smith H.E. (2016). Correlations between quantitative T2 and T1ρ MRI, mechanical properties and biochemical composition in a rabbit lumbar intervertebral disc degeneration model. J. Orthop. Res..

[45] Berberan-Santos M.N., Bodunov E.N., Valeur B. (2005). Mathematical functions for the analysis of luminescence decays with un-derlying distributions. Kohlrausch decay function (stretched exponential). Chem. Phys..

[46] Van der Veen A.J., Bisschop A., Mullender M.G., van Dieën J.H. (2013). Modelling creep behaviour of the human intervertebral disc. J. Biomech..

[47] Yang M.Y., Xiang D.D., Chen Y.R., Cui Y.Y., Wang S., Liu W.Q. (2022). An Artificial PVA-BC Composite That Mimics the Biomechanical Properties and Structure of a Natural Intervertebral Disc. Materials.

[48] Beckstein J.C., Sen S., Schaer T.P., Vresilovic E.J., Elliott D.M. (2008). Comparison of animal discs used in disc research to human lumbar disc: Axial compression mechanics and glycosaminoglycan content. Spine.

[49] Oxland T.R., Panjabi M.M., Southern E.P., Duranceau J.S. (1991). An anatomic basis for spinal instability: A porcine trauma model. J. Orthop. Res..

[50] Yingling V.R., Callaghan J.P., McGill S.M. (1999). The porcine cervical spine as a model of the human lumbar spine: An anatomical, geometric, and functional comparison. J. Spinal Disord..

[51] Chan S.C.W., Gantenbein-Ritter B., Leung V.Y.L., Chan D., Cheung K.M.C., Ito K. (2010). Cryopreserved intervertebral disc with injected bone marrow-derived stromal cells: A feasibility study using organ culture. Spine J..

[52] Miles D.E., Mitchell E.A., Kapur N., Beales P.A., Wilcox R.K. (2016). Peptide: Glycosaminoglycan hybrid hydrogels as an injectable intervention for spinal disc degeneration. J. Mater. Chem. B.

[53] Hom W.W., Tschopp M., Lin H.A., Nasser P., Laudier D.M., Hecht A.C. (2019). Composite biomaterial repair strategy to restore biomechanical function and reduce herniation risk in an ex vivo large animal model of intervertebral disc herniation with varying injury severity. PLoS ONE.

[54] Sapiee N.H., Thambyah A., Robertson P.A., Broom N.D. (2019). Sagittal alignment with downward slope of the lower lumbar motion segment influences its modes of failure in direct compression: A mechanical and microstructural investigation. Spine.

[55] Feki F., Taktak R., Kandil K., Derrouiche A., Moulart M., Haddar N., Zaïri F., Zaïri F. (2020). How osmoviscoelastic coupling affects recovery of cyclically compressed intervertebral disc. Spine.

[56] Derrouiche A., Feki F., Zaïri F., Taktak R., Moulart M., Qu Z., Ismail J., Charfi S., Haddar N., Zaïri F. (2020). How pre-strain affects the chemo-torsional response of the intervertebral disc. Clin. Biomech..

[57] Werbner B., Spack K., O’Connell G.D. (2019). Bovine annulus fibrosus hydration affects rate-dependent failure mechanics in tension. J. Biomech..

[58] Tavakoli J., Diwan A.D., Tipper J.L. (2020). Elastic fibers: The missing key to improve engineering concepts for reconstruction of the nucleus pulposus in the intervertebral disc. Acta Biomater..

[59] Du Y., Tavana S., Rahman T., Baxan N., Hansen U.N., Newell N. (2021). Sensitivity of intervertebral disc finite element models to internal geometric and non-geometric parameters. Front. Bioeng. Biotechnol..

[60] Tamoud A., Zaïri F., Mesbah A., Zaïri F. (2022). A fully three-dimensional model of interpenetrating collagen fibrillar networks for intervertebral disc mechanics. Int. J. Mech. Sci..

[61] Yoon D.H.E., Weber C.I., Easson G.W.D., Broz K.S., Tang S.Y. (2020). Rapid determination of internal strains in soft tissues using an experimentally calibrated finite element model derived from magnetic resonance imaging. Quant. Imaging Med. Surg..

[62] Komeili A., Rasoulian A., Moghaddam F., El-Rich M., Li L.P. (2021). The importance of intervertebral disc material model on the prediction of mechanical function of the cervical spine. BMC Musculoskelet. Disord..

[63] Castro A.P.G., Alves J.L. (2020). Numerical implementation of an osmo-poro-visco-hyperelastic finite element solver: Application to the intervertebral disc. Comput. Methods Biomech. Biomed. Eng..

[64] Subramani A.V., Whitley P.E., Garimella H.T., Kraft R.H. (2020). Fatigue damage prediction in the annulus of cervical spine intervertebral discs using finite element analysis. Comput. Methods Biomech. Biomed. Eng..

[65] Kandil K., Zaïri F., Messager T., Zaïri F. (2021). A microstructure-based model for a full lamellar-interlamellar displacement and shear strain mapping inside human intervertebral disc core. Comput. Biol. Med..

